# Electrochemical Sensing of Metribuzin Utilizing the Synergistic Effects of Cationic and Anionic Bio-Polymers with Hetero-Doped Carbon

**DOI:** 10.3390/polym17010039

**Published:** 2024-12-27

**Authors:** Thirukumaran Periyasamy, Shakila Parveen Asrafali, Seong-Cheol Kim, Jaewoong Lee

**Affiliations:** 1 Department of Fiber System Engineering, Yeungnam University, Gyeongbuk 38541, Republic of Korea; thirukumaran@ynu.ac.kr (T.P.); shakilaparveen@yu.ac.kr (S.P.A.); 2 School of Chemical Engineering, Yeungnam University, Gyeongbuk 38541, Republic of Korea; sckim07@ynu.ac.kr

**Keywords:** polybenzoxazine-based carbon, amino cellulose, hyaluronic acid, bio-polymer complex, metribuzin detection

## Abstract

The development of innovative, cost effective, and biocompatible sensor materials for rapid and efficient practical applications is a key area of focus in electroanalytical chemistry. In this research, we report on a novel biocompatible sensor, made using a unique polybenzoxazine-based carbon combined with amino cellulose and hyaluronic acid to produce a bio-polymer complex (PBC-ACH) (polybenzoxazine-based carbon with amino cellulose and hyaluronic acid). This sensor material is fabricated for the first time to enable the electroreduction of the herbicide, metribuzin (MTZ). The PBC-ACH sensor presents multiple advantages, including ease of fabrication, excellent biocompatibility, and low-cost production, making it suitable for various applications. In optimized experimental conditions, the sensor was fabricated by modifying a glassy carbon electrode (GCE) with the PBC-ACH complex, resulting in the creation of a GCE/PBC-ACH electrode. This modified electrode demonstrated the ability to detect MTZ at nanomolar levels, with an LoD of 13.04 nM, showcasing a high sensitivity of 1.40 µA µM^−1^ cm^−2^. Moreover, the GCE/PBC-ACH sensor exhibited remarkable selectivity, stability, and reproducibility in terms of its electrochemical performance, which are essential features for reliable sensing applications. The potential mechanism behind the detection of MTZ using the GCE/PBC-ACH sensor was investigated thoroughly, providing insights into its sensing behavior. Additionally, tests on real samples validated the sensor’s practicality and efficiency in detecting specific analytes. These findings emphasize the potential of the GCE/PBC-ACH sensor as a highly effective electrochemical sensor, with promising applications in environmental monitoring and other fields requiring precise analyte detection.

## 1. Introduction

As a result of agricultural activities, the release of herbicides into the environment causes substantial health risks to human beings and aquatic animals. Owing to their poisonous nature, there is growing concern about the need to establish a reliable analytical procedure to estimate the content of herbicides in agricultural products, such as crops, fruits and vegetables, soil, and water [1,2]. Metribuzin (MTZ) is an asymmetrical triazine herbicide that is widely used in crops, such as sugarcane, tomatoes, potatoes, soybeans, carrots, asparagus, maize, and cereals, to control the growth of grasses and weeds [3,4]. MTZ is considered to be a Group D carcinogen, which means that its toxicological effects are weaker in humans, but hazardous to aquatic animals like fish, invertebrates, freshwater macrophytes, algae, and aquatic plants. MTZ can easily infiltrate soil profiles, surface water, and ground water due to its high solubility in water (1050 mg L^−1^ at 20 °C), low octanol–water partition coefficient (K_ow_ = 44.7), and low soil organic carbon partition coefficient (K_oc_ = 3.14–81.5 mL g^−1^) [5,6,7,8]. And so, a simple and effective method for the detection of metribuzin is required. The electrochemical method is preferred over analytical techniques (such as capillary electrophoresis, the flow injection system, voltammetry, and gas chromatography–mass spectrometry), due to its sensitivity, accompanied by its selectivity, precision, portability, and cost effectiveness [9,10,11,12,13,14]. In this regard, progress in terms of the rational design and construction of materials possessing novel properties is urgently needed.

Presently, the development of electrochemical sensors, based on sustainable naturally derived bio-polymers and bio-macromolecules, has attracted research interest, as they confer several advantages over synthetic materials, including biocompatibility, biodegradability, and non-toxicity [3,15,16,17]. It has been reported that composite materials can improve the sensitivity of electrochemical sensors by producing synergistic electrocatalytic activity. Several composite materials have been synthesized by incorporating noble metals, semiconductor nanoparticles, and ionic liquids, conducting polymers and bio-polymers on the surface of graphene, with applications in several fields, including energy storage, electrocatalysis, and use in biosensors. In recent years, polyelectrolytes (PEs) derived from bio-polymers have exhibited superior performance in electrochemical sensing. Instead of using a single polymer, a combination of polymers improves the sensitivity of the electrode through their synergistic effect. Blending polycationic bio-polymers, such as chitosan, cellulose, starch, and dextran, etc., with polyanionic bio-polymers, such as alginate, pectin, carrageenan, xanthan gum, and carboxymethylcellulose, etc., is an effective way to produce a bio-polymer-based polyelectrolyte [18,19,20,21,22].

So far, there are a few reports on chitosan-based PEs in electrochemical sensing. Rassas et al. [19], in 2019, designed a voltammetric glucose biosensor, using a chitosan/k-carrageenan polyelectrolyte complex. The fabricated biosensor exhibited a wide linearity range for glucose detection from 5 µM to 7 mM, with an LoD of 5 µM (LoD = limit of detection). In the same year, Ranganathan et al. [3] developed a novel biocompatible sensor, CS-PC BPE (chitosan–pectin bio-polyelectrolyte), for the simultaneous detection of metronidazole and metribuzin. The sensing ability of the CS-PC BPE/GCE (glassy carbon electrode) was found to be in the nM range, possessing prominent selectivity, stability, and reproducibility. Recently in 2024, Hsiao et al. [23] prepared a carrageenan-derived polyelectrolyte for the electrochemical sensing of sulfamethazine. The k-CGN/P(Am-co-DMDAAc)-GO [k-carrageenan/poly (acrylamide-co-dimethyl diallyl ammonium chloride)]-graphene oxide-modified GCE has excellent detection ability in the nM range, with an anti-interference ability.

Carbon materials have been used in applications in several fields, including energy storage, sensors, electrocatalysis, and so on. They have varied structures, from zero to three dimensions, and are abundant in the earth, with a low cost. Moreover, they possess a high surface area, with tunable surface modification of heteroatom doping [24,25,26,27]. We produced carbon materials from a polybenzoxazine source, which are considered to be an advanced form of phenolic resins. Their advantage in terms of their molecular design flexibility can be utilized to arrive at the desired molecular structure. This in turn results in the formation of carbon atoms with the desired amount of doped heteroatoms [28].

The novelty of this work lies in the fabrication of a bio-polymer composite material consisting of hetero-doped carbon and polycationic and polyanionic bio-polymers. Here, polybenzoxazine acts as a source to produce hetero-doped carbon. The heteroatoms are occupied in the interstices when produced from a polybenzoxazine origin and they cannot be easily removed from the carbon framework. Amino cellulose, a derivative of cellulose, which is the principle ingredient in plant-based biomass, acts as the polycationic bio-polymer. Hyaluronic acid, also known as hyaluronan, a non-sulfated glycosaminoglycan, acts as the polyanionic bio-polymer. The combination of polyanionic and polycationic bio-polymers delivers a synergistic electrochemical sensing property and hetero-doped carbon adds conductivity to the bio-polymer composite.

## 2. Materials and Methods

### 2.1. Materials

Melamine, cellulose, eugenol, hyaluronic acid, and paraformaldehyde were purchased from Sigma-Aldrich (St. Louis, MO, USA). N,N-dimethylacetamide (DMAc), sodium hydroxide (NaOH), anhydrous LiCl, acetic acid, dimethyl sulfoxide (*DMSO*), *p*-toluenesulfonyl chloride, and ethylene diamine were purchased from Duksan Chemicals Co., Ltd., Ansan, Republic of Korea. Ethanol was purchased from Daejung Chemicals Co., Ltd., Siheung-si, Republic of Korea.

### 2.2. Synthesis of PBC-ACH Complex

**Scheme 1:**
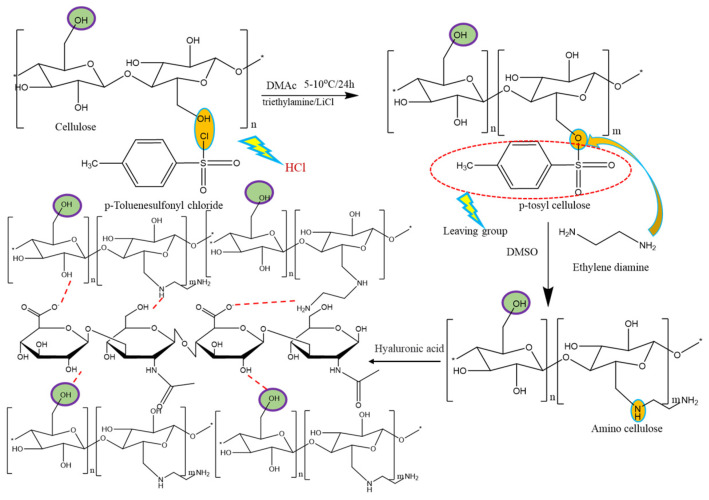
Scheme showing the formation of amino cellulose and bio-polymer complex (ACH).

The synthesis of polybenzoxazine-based carbon (PBC) and amino cellulose (AC), and the instrumentation and characterization of PBC-ACH are explained in the Supporting Information. From our previous work [28], we found that a calcination temperature of 800 °C and activation with KOH is effective for preparing PBC with a high specific surface area of 984 m^2^ g^−1^, containing a micro- and mesoporous structure, with a maximum number of pores in the mesoporous range (2–50 nm), and nitrogen content of 6.26 wt.% and oxygen content of 13.65 wt.%. This PBC will be effective in regard to electrochemical sensing when used in a complex with bio-polymer(s). The PBC-ACH complex was synthesized by modifying the procedures described by Bigucci et al. and Maciel et al. [29,30]. Separately, amino cellulose (AC) and hyaluronic acid were dissolved in a 10% acetic acid solution. Then, 25 mL of the AC solution was mixed with 25 mL of the hyaluronic acid solution. Following this, 15 wt.% of PBC was added and the mixture was stirred at room temperature for 24 h. The resulting PBC-ACH precipitate was isolated using ultracentrifugation at 6000 rpm for 30 min at a temperature of 5 ± 1 °C. The precipitate was washed with double-distilled water and dried under a vacuum at 40 °C for 12 h. Finally, the dried precipitate was re-dispersed in water for use in the subsequent experiments. Scheme 1 illustrates the synthesis process for the PBC-ACH complex. Figure S1 shows the FT-IR spectra of cellulose, tosyl cellulose, amino cellulose, hyaluronic acid and ACH.

### 2.3. Fabrication of GCE/PBC-ACH

**Scheme 2:**
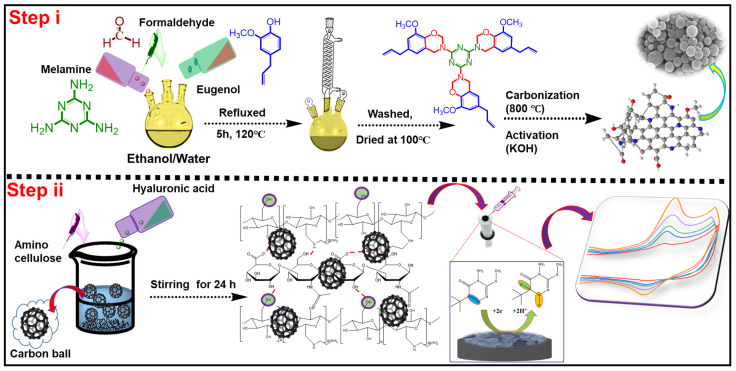
Schematic representation showing the synthesis procedure for PBC (Step i) and PBC-ACH (Step ii).

Before modification, the glassy carbon electrode (GCE) was thoroughly polished using 0.05 μm alumina powder and then rinsed with double-distilled (DD) water. The prepared PBC-ACH solution (1 mL) was mixed with an equal volume (1 mL) of DD water, with a ratio of 1:1, and subjected to ultra-sonication to ensure uniform dispersion. Next, 4 μL of this PBC-ACH solution was drop cast onto the surface of the GCE and allowed to dry at room temperature. The resulting modified electrode (GCE/PBC-ACH) was then used for electrochemical measurements. Scheme 2 illustrates the synthesis process for the PBC-ACH complex and the electrochemical reduction mechanism of MTZ [29,30].

## 3. Results and Discussion

### 3.1. SEM Analysis

**Figure 1:**
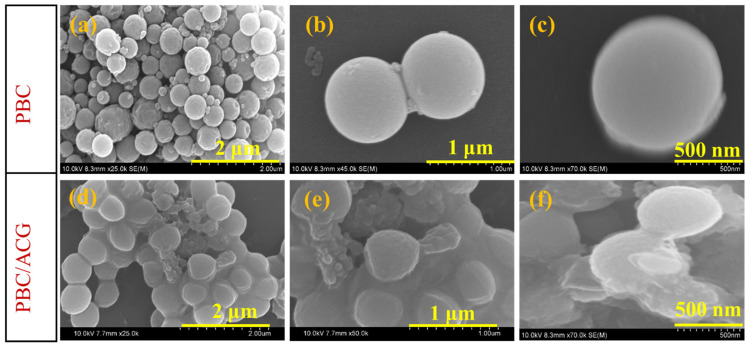
SEM images of (**a**–**c**) PBC and (**d**–**f**) PBC-ACH at different magnifications.

The morphology of PBC and PBC-ACH was analyzed using SEM images. The SEM images of PBC (Figure 1a–c) reveal the highly uniform distribution of spherical carbon particles. These spheres appear to be well-defined, with smooth and consistent surfaces. The sizes of these spheres seem to be relatively homogeneous, indicating controlled synthesis. At higher magnification (Figure 1c), the detailed structure of the individual spheres becomes clearer. The surface remains smooth and the spheres retain their shape, even at higher resolutions. There is minimal agglomeration, suggesting good stability and dispersion. The introduction of another component, ACH, significantly alters the morphology of the spheres (Figure 1d–f). The images show less uniformity in the shape and size of the particles, with some spheres appearing to be deformed or partially merged with one another. The surface of the spheres in the PBC-ACH complex appears to have an irregular morphology, rough surfaces, and more pronounced agglomeration when compared with PBC spheres. This could be due to interactions between the carbon spheres of PBC and ACH, leading to partial coalescence or surface coating. At higher magnification (Figure 1f), the rough surface texture becomes more evident. The spheres also show signs of partial integration with the surrounding material, suggesting possible chemical or physical bonding between PBC and ACH. These observations are crucial in determining the suitability of these materials for specific applications, such as in composites, coatings, or as conductive materials. Figure S2 shows the XPS spectrum of PBC-ACH.

### 3.2. TEM Analysis

**Figure 2:**
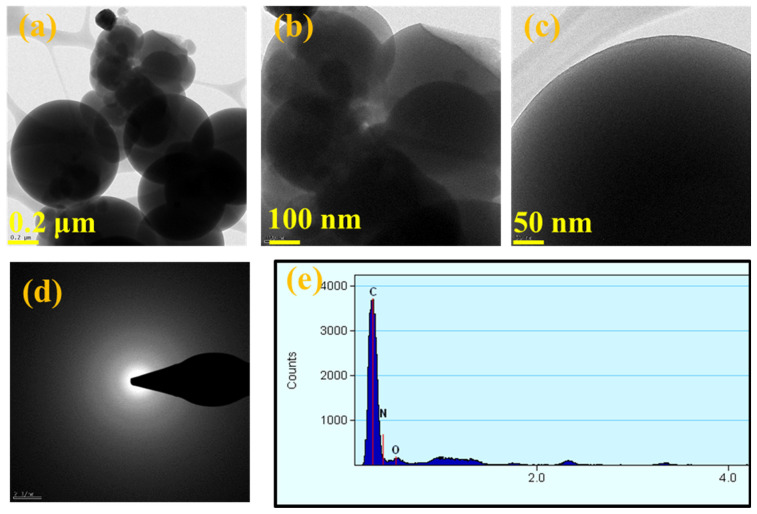
TEM images of PBC-ACH at different magnifications (**a**–**c**), SAED pattern (**d**), and EDX spectrum (**e**).

TEM analysis was carried out to provide deeper insight into the morphology of PBC-ACH and the images are displayed in Figure 2a–c. The low-magnification TEM image (Figure 2a) reveals a cluster of polymer-coated carbon spheres. The spheres appear to be well-dispersed, with a consistent shape and size, indicating a uniform coating. The ACH seems to be evenly distributed around each carbon core. An image with higher magnification of an individual carbon ball is shown in Figure 2c. The polymer coating is clearly visible, encapsulating the carbon core. The coating appears to be uniform in terms of thickness, suggesting a controlled deposition process. The smooth interface between the polymer and carbon core is indicative of strong adhesion between the two materials. The polymer coating exhibits a distinct and well-defined structure, highlighting its nanoscale thickness. The carbon core appears to be dense and uniform, with no apparent defects or irregularities. Figure 2d represents a Selected-Area Electron Diffraction (SAED) pattern, showing the crystalline structure of the carbon core. The pattern indicates that the carbon balls are either amorphous or exhibit limited crystallinity, which is common for polymer-coated carbon materials. The EDX spectrum in Figure 2e highlights the elemental composition of the material, showing the peaks corresponding to carbon (C), nitrogen (N), and oxygen (O) elements. The dominance of the carbon peak confirms the presence of carbon as the primary component, while the nitrogen and oxygen peaks are likely from the ACH coating on the surface of the PBC. The combination of SEM and TEM analyses provides comprehensive insights into the material’s morphology and composition, which are essential for applications in areas related to electrochemical study.

### 3.3. Electrochemical Results

#### 3.3.1. Electrochemical Performance of the Prepared Electrodes

**Figure 3:**
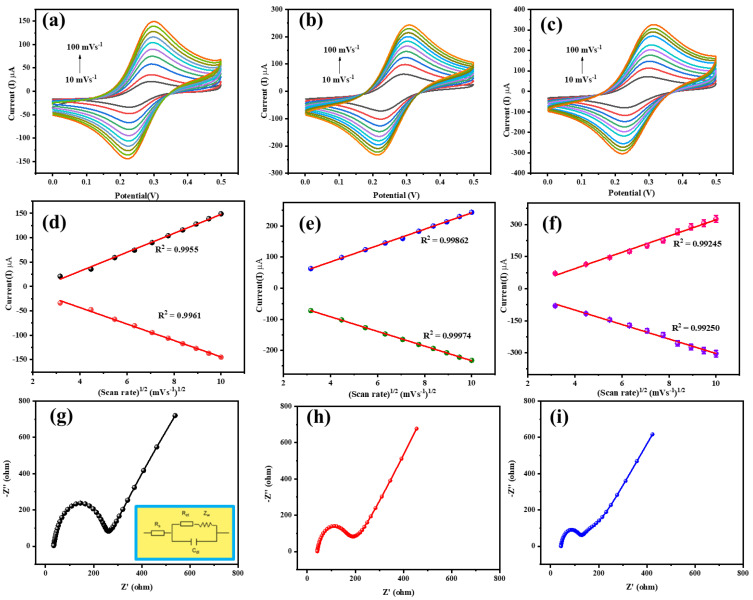
Electrochemical studies of bare GCE, GCE/PBC, and GCE/PBC-ACH: (**a**–**c**) CV graphs at different scan rates, (**d**–**f**) linear plots of scan rate vs. peak current, and (**g**–**i**) Nyquist plots.

The electrochemical performance of the prepared electrodes, bare GCE, GCE/PBC, and GCE/PBC-ACH was scrutinized using CV in an electrolytic solution containing 0.5 M KCl, with 5 mM of K_3_[Fe(CN)_6_]/K_4_[Fe(CN)_6_], at a scan rate of 50 mVs^−1^. Well-defined redox peaks were obtained (Figure 3a–c) for all the electrodes, indicating a reversible charge transfer process. The anodic and cathodic peak currents (I_pa_ and I_pc_) were found to be I_pa_ = 93.75 µA and I_pc_ = −91.67 µA for the bare GCE; I_pa_ = 162.05 µA and I_pc_ = −162.5 µA for the GCE/PBC; and I_pa_ = 199.05 µA and I_pc_ = −198.2 µA for the GCE/PBC-ACH. In addition to this, the peak-to-peak separation (ΔE_p_) was found to be 0.065, 0.08, and 0.06 V for the bare GCE, GCE/PBC, and GCE/PBC-ACH, respectively. The results show that the enhanced redox peak current and lower ΔE_p_ value of GCE/PBC-ACH are due to the presence of a large surface area and abundant active sties, which improved its electron transfer ability when compared with the other two electrodes [1,3,31].

The electron transfer properties of the bare GCE, GCE/PBC, and GCE/PBC-ACH electrodes were studied using EIS [Electrochemical Impedance Spectroscopy], in the frequency range between 100 MHz and 1 kHz, using the same electrolytic solution {0.5 M KCl containing 5 mM K_3_[Fe(CN)_6_]/K_4_[Fe(CN)_6_]}. Figure 3g–i displays the Nyquist plots of all the electrodes, showing a semicircle in the high frequency region and a linear graph in the low frequency region. The diameter of the semicircle in the high frequency region indicates charge transfer resistance (R_ct_), which is attributed to the kinetically controlled electron transfer. The linear portion in the low frequency region indicates Warburg resistance (Z_w_), which is attributed to the mass transfer. The Randles fitting equivalent circuit model is used for EIS data interpolation (Table 1). The solution resistance (R_s_) was found to be 76 Ω for the bare GCE; 100 Ω for the GCE/PBC; and 105 Ω for the GCE/PBC-ACH. It was found that the bare GCE possessed an R_ct_ value of 233 Ω, indicating the sluggish transfer of electrons. Whereas the GCE/PBC possessed a low R_ct_ value of 162 Ω and the GCE/PBC-ACH possessed a very low R_ct_ value of 89 Ω, which manifested as the rapid electron transfer ability of GCE/PBC-ACH, with a highly accessible surface area [31,32,33]. In addition to this, a straight line inclined at an angle of 45° was observed soon after the semicircle for all the electrodes. This indicates that the electrochemical reaction is governed by the diffusion of the electrolyte species.

The detailed electrocatalytic activity of the electrodes was measured by employing CV, with increasing scan rates from 10 to 100 mVs^−1^, in the potential range from 0 to 0.5 V. Figure 3a–f shows the CV of the electrodes and their corresponding linear plots of the scan rate and redox peak currents. With increasing scan rates, there is a gradual increase in the redox peak currents for all the three electrodes. The linear regression equation obtained from the plots of the scan rate and redox current peaks was used to calculate the electrochemically active surface area. The Randles–Sevcik equation was used:

I_pa_ = 2.69 × 10^5^ AD^1/2^ n^3/2^ ν^1/2^ C (1)

where I_pa_ is the anodic peak current, A is the electroactive surface area, D is the diffusion coefficient, *n* is the number of electrons transferred, ν is the scan rate, and C is the concentration of K_3_[Fe(CN)_6_]/K_4_[Fe(CN)_6_]. The electrochemically active surface area was calculated to be 0.0568 cm^2^ for the bare GCE; 0.0778 cm^2^ for the GCE/PBC; and 0.098 cm^2^ for the GCE/PBC-ACH. In comparison, the GCE/PBC-ACH possessed a large active surface area, enabling rapid electron transfer, which could promote efficient detection performance of the prepared electrode towards metribuzin [34,35,36].

#### 3.3.2. Electrochemical Behavior of the Prepared Electrodes in Regard to MTZ Detection

**Figure 4:**
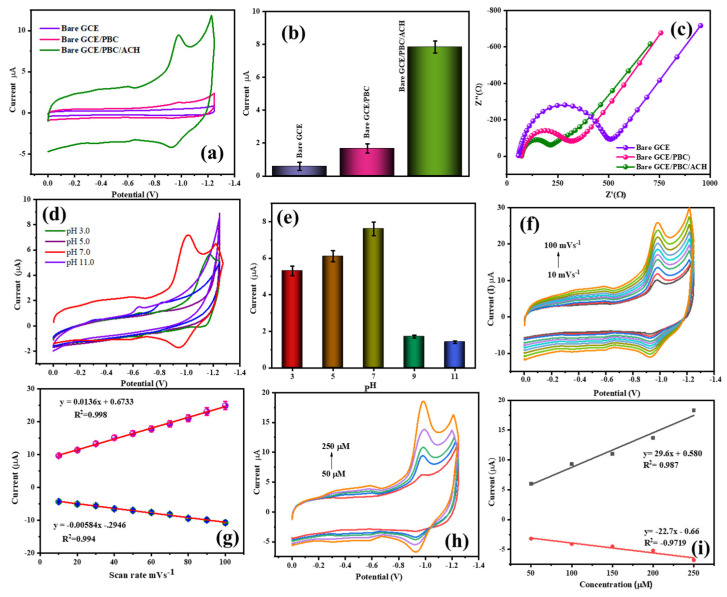
Bare GCE, GCE/PBC, and GCE/PBC-ACH: (**a**,**b**) CV graphs and peak current values showing the detection of MTZ, (**c**) Nyquist plots. GCE/PBC-ACH: detection of MTZ (**d**,**e**) at different pH and peak current values, (**f**,**g**) at different scan rates and the linear plots, and (**h**,**i**) at different GCE/PBC-ACH concentrations and the linear plots.

The electrochemical reduction of MTZ using the bare GCE, GCE/PBC, and GCE/PBC-ACH was recorded using CV, at a scan rate of 50 mVs^−1^. Phosphate buffer (pH = 7) acts as the electrolytic medium, to which 30 µM of MTZ was added, and the CV was monitored in the negative potential range between 0 and −1.2 V, and the results are depicted in Figure 4a. The current peak obtained for each electrode is plotted in Figure 4b. As can be observed, the bare GCE had a diminished redox peak, with a peak current well below 1 µA. Whereas, in comparison, the GCE/PBC showed a slight increase in its peak current value, to about 1.25 µA. Comparatively, the modified electrode, GCE/PBC-ACH showed a well-defined redox peak, with higher peak current values of I_pa_ = 7.9 µA and I_pc_ = −4.37 µA and an ΔE_p_ of 0.06 V. This increment towards the electrochemical detection of MTZ is due to the active surface area and mesoporous structure of the GCE/PBC-ACH electrode that paves way for the creation of many approachable active sites. The impedance spectra for all the electrodes are shown in Figure 4c. The charge transfer resistance obtained from the Nyquist plots (Figure 4c) were found to be 437.5 Ω for the bare GCE; 250 Ω for the GCE/PBC; and 145 Ω for the GCE/PBC-ACH. This further confirms the enhanced conductivity of GCE/PBC-ACH.

#### 3.3.3. Effect of pH

The current response of GCE/PBC-ACH towards 30 µM MTZ at a varying pH, from 3 to 11, was analyzed using CV and the results are depicted in Figure 4d. For pH 3, citric acid/NaOH/HCl was used; for pH 5 and 7, 0.1 M phosphate buffer was used; for pH 9 and 11, boric acid/KCl/NaOH was used. A well-defined redox peak, with an increased peak current at 7.9 µA, could be observed at pH = 7. The calibration plot (Figure 4e) of the pH and peak current reveals the fact that a gradual increase in the peak current was observed from pH 3 to pH 7, and with a further increase in the pH values (pH 9 and pH 11), there is a drastic decrease in the peak current values. This indicates that the pH of the electrolytic solution has control over the electroreduction mechanism of MTZ by GCE/PBC-ACH. At pH = 7, the protonation and deprotonation of MTZ species dominates, which results in an increase in the current peak intensity. Hence, pH 7 was maintained for further electrochemical studies [1,3,31,37,38].

#### 3.3.4. Effect of the Scan Rate

To gain a deeper understanding of the electrochemical mechanism of MTZ, CV analysis at varying scan rates, from 10 to 100 mVs^−1^, was performed and the results from the electrochemical detection of MTZ by GCE/PBC-ACH are displayed in Figure 4f. It is clearly evident that increasing the scan rate results in an increase in the peak current in regard to both the oxidation and reduction potential. A good linear fit between the redox peak current and the scan rate was observed (Figure 4g), and the linear regression equation is given by y = 0.0136x + 0.6733 (R^2^ = 0.998) for the anode and y = −0.00584x − 2.946 (R^2^ = 0.994) for the cathode. This indicates that the electrochemical response is monitored by a surface-controlled process. Moreover, the reduction peak potential shifted towards the more negative region with an increase in the scan rate, indicating a kinetic limitation during electrochemical reduction. Furthermore, the slope value of the regression equation was used to calculate the α value (charge transfer coefficient), applying the Butler–Volmer equation given below [39,40]:

E_pa_ = E_0_ − (RT/αnF) ln (RTk_s_/αnF) + RT/2αnF ln ν (2)

From Equation (2), RT/2αnF represents the slope, which is found to be 0.0136 (from the regression equation). By substituting the values for R (gas constant), T (room temperature), *n* (number of electrons transferred, *n* = 2 for MTZ), and F (Faraday’s constant), the alpha value was found to be 0.472, indicating an irreversible electrochemical detection process.

### 3.4. Material Concentration

To study the effect of the electrode material concentration on MTZ detection five different concentrations of PBC-ACH, from 50 to 250 µM, were used to coat the GCE, and the CV was analyzed. The CV curves obtained are displayed in Figure 4h and the linear plots of the anodic and cathodic peak currents, with respect to the PBC-ACH concentration, are displayed in Figure 4i. A prominent redox peak was observed at 100 µM of PBC-ACH. The intensity of the redox peak reduced further with an increase in the concentration of PBC-ACH. This shows that the thickness of the PBC-ACH used to coat the GCE, of up to 100 µM, was effective, and as the thickness of the electrode increases (i.e., an increasing concentration of PBC-ACH), it reduces the charge transfer efficiency and affects the electrochemical detection process.

### 3.5. Analyte Concentration

**Figure 5:**
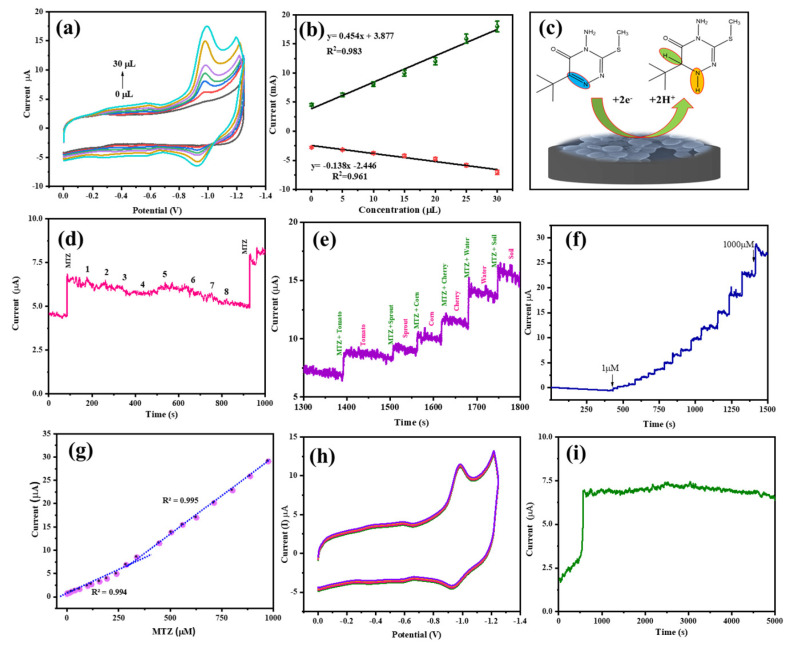
(**a**,**b**) CV graphs showing the electrochemical detection of MTZ by GCE/PBC-ACH at different analyte concentrations and the linear plots, (**c**) electrochemical reduction mechanism of MTZ, (**d**–**i**) anti-interfering ability, repeatability, reproducibility, and stability of GCE/PBC-ACH.

The electrocatalytic activity of GCE/PBC-ACH in 0.1 M phosphate buffer with different concentrations of the analyte, MTZ (0.01 to 30 µM), was studied using CV analysis, at a scan rate of 50 mVs^−1^. The obtained CV curves and the linear plots of the current peak vs. the MTZ concentration are presented in Figure 5a,b. There is a linear increase in the cathodic and anodic peak current with an increase in the concentration of MTZ, illustrating the excellent electrocatalytic activity of the prepared electrode material. This enhanced electrocatalytic activity is due to (i) the proper combination of the polycationic amino cellulose group and the polyanionic hyaluronic acid group; (ii) the use of the heteroatom-derived carbon material from polybenzoxazine (PBC) to provide a large surface area that could increase the adsorption of MTZ and to maintain the stability of the electrode; (iii) the electrostatic attraction between PBC and amine, imine, and carbonyl groups of MTZ; and (iv) the hydrogen bonding interactions between –NH_2_ and –C=O groups of MTZ with –OH, –NH_2_, COO^−^ groups of amino cellulose and hyaluronic acid, which accelerates the electron transfer kinetics of MTZ through PBC-ACH [29,35]. As the detection of MTZ proceeds through a reduction reaction, the slope value obtained from the regression equation in regard to the cathodic region was used to calculate the LoD (limit of detection), LoQ (Limit of Quantitation), and sensitivity, using the equations given below:

LoD = 3 sb/s (3)

LoQ = 10 sb/s (4)

Sensitivity = slope/active surface area(5)

where ‘sb’ denotes the current obtained for the blank solution without the analyte and ‘s’ denotes the slope value obtained from the regression equation. The calculated values for the LoD, LoQ, and sensitivity were found to be 0.217 µM, 0.724 µM, and 1.40 µA µM^−1^ cm^−2^. The results confirm that the increased electrochemically active surface area, the excellent conductivity of PBC-ACH, and the innumerable active sites pave the way for the effective detection of MTZ.

### 3.6. Anti-Interference Property

The anti-interference ability of GCE/PBC-ACH towards different metal ions was analyzed in 0.1 M phosphate buffer, containing 20 µM of MTZ, and the current peaks obtained are displayed in Figure 5d. The interfering metal ions, such as Ag^+^, K^+^, Li^+^, Na^+^, Zn^2+^, Ca^2+^, Mg^2+^, and Ni^2+^, with a concentration of 1000 µM, were added separately, at a particular time interval, and the change in the current peak was noted. Before the addition of the metal ions, MTZ shows a current peak at around 7.2 µM. The addition of the metal ions showed a slight variation in the current peaks, with a deviation of around 5%, and again, after the addition of 20 µM MTZ, the current peak increased further. This clearly shows that the interfering metal ions, with almost a 50-fold increase in concentration, do not show any obvious influence on the current peaks. Similarly, the anti-interference ability of GCE/PBC-ACH towards real samples was also validated and the results are displayed in Figure 5e. An increase in the current peak was observed whenever MTZ was added, but with the addition of real samples, there was no increase in the current peak. Both of these results suggest that the GCE/PBC-ACH sensor has excellent anti-inference properties.

### 3.7. Repeatability, Reproducibility, and Stability

The repeatability of the GCE/PBC-ACH sensor was evaluated by adding different concentrations of MTZ, from 1 µM to 1000 µM, to the phosphate buffer solution and measuring the current for a certain period of time for each MTZ addition (Figure 5f,g). There is an increase in the current peak for each increase in MTZ concentration. But, when each MTZ addition was prolonged for some time, the current peak remains constant. To check the reproducibility of the prepared sensor, five different electrodes using GCE/PBC-ACH were fabricated with the same procedure and the CV was measured by injecting 30 µM of the analyte (Figure 5h). The redox peak currents were noted from the CV graph. The CV curves do not show much deviation in the redox peaks for the five different sensors. The two abovementioned methods elucidate acceptable repeatability and reproducibility in terms of the prepared GCE/PBC-ACH sensor. In addition to this, the stability of the sensor was analyzed by measuring the current peak for a period of 5000 s (Figure 5i). The current value was reduced slightly from the initial current value, retaining about 93.9% of the initial current value. This result proves that GCE/PBC-ACH sensor has excellent stability.

### 3.8. Real-Time Application

**Figure 6:**
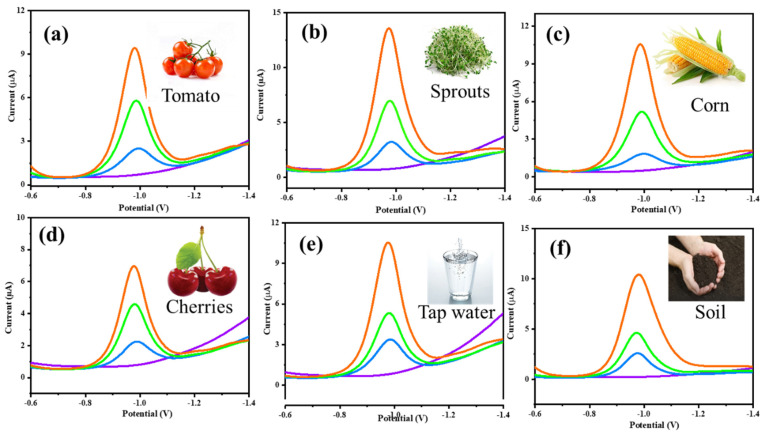
(**a**–**f**) DPV graphs showing the detection of MTZ in real samples.

The accuracy of the prepared sensor was estimated by performing a recovery experiment. Real samples, such as cherry tomatoes, sprouts, corn, berries, tap water, and soil, were used in the recovery experiment. Except for tap water and soil, all the other samples are crushed, filtered, and their extract was used for the experiment. The tap water was used as is, and, in the case of soil, it was added to water, mixed well, filtered, and the soil water was used. A known concentration of MTZ (say 5, 7, and 10 µM) was added to the real samples and a DPV measurement was conducted. Figure 6a–f shows the DPV curves, indicating the current obtained for the different samples with different concentrations of MTZ. A linear increase in the current peak was obtained with an increase in the concentration of MTZ [1,3,31]. The fabricated GCE/PBC-ACH sensor exhibits excellent recovery rates in the range of 97.89–103.37%, along with low RSD values of 0.36–2.65%, widening the potential applicability of GCE/PBC-ACH in regard to real-time applications. A comparison of the electrochemical sensors used in the detection of metribuzin is presented in Table 2.

## 4. Conclusions

A novel, environmentally friendly method has been developed for synthesizing biocompatible PBC-ACH, which has been successfully applied for the first time in the electrochemical detection of the herbicide, metribuzin. The PBC-ACH was straightforwardly deposited onto a glassy carbon electrode (GCE) via a simple drop-casting technique, which significantly enhanced both the electron transfer efficiency and the electrocatalytic activity of the sensor. This uncomplicated preparation process resulted in a highly sensitive MTZ sensor, characterized by a low LoD of 13.04 nM and a low LoQ of 43.47 nM, with a sensitivity of 1.04 μA M^−1^ cm^−2^. The GCE/PBC-ACH system, by utilizing differential pulse voltammetry, determined the detection of MTZ in a variety of real-world samples, including tomatoes, sprouts, corn, cherries, tap water, and soil, with satisfactory outcomes. This type of biocompatible electrochemical sensor demonstrates outstanding analytical performance, without relying on any metal nanoparticles, making it a competitive alternative in the field of environmental monitoring. Additionally, the remarkable biocompatibility and biodegradability of this electrochemical sensor align well with the principles of sustainable development and the circular economy. The innovative approach described here presents a promising avenue for the future design and application of green, biocompatible sensors for environmental analysis.

## Figures and Tables

**Table 1:** Data obtained from EIS measurements.

Electrode	R_s_ (Ω)	R_ct_ (Ω)	Z_w_ (Ω)
Bare GCE	76	233	724
GCE/PBC	100	162	680
GCE/PBC-ACH	105	89	616

**Table 2:** A comparison of the electrochemical sensors used in the detection of metribuzin.

Sensor Material	Electrode Type	Detection Technique	Linear Range	Limit of Detection (LoD)	Sensitivity	Reference
Chitosan–Pectin Bio-Polyelectrolyte (CS-PC BPE)	GCE	Voltammetry	nM range	N/A	High selectivity and stability	[3]
LaFeO3/RGO Nanocomposite	Modified Electrode	Voltammetry	N/A	N/A	Sensitive detection in real samples	[9]
Multilayer Graphene–Gold Nanoparticle	Modified Electrode	Surface-Enhanced Raman Scattering (SERS)	N/A	N/A	High sensitivity for low concentrations	[10]
Chitosan/K-Carrageenan Polyelectrolyte	Glassy Carbon Electrode (GCE)	Voltammetry	5 µM–7 mM	5 µM	N/A	[19]
K-Carrageenan-Derived Polyelectrolyte (k-CGN/P(Am-co-DMDAAc)-GO)	GCE	Electrochemical Sensing	nM range	N/A	Excellent anti-interference ability	[23]
PBC-ACH Composite (Current Study)	GCE/PBC-ACH	Cyclic Voltammetry (CV)	0.01–30 µM	0.217 µM	1.40 µA µM^−1^ cm^−2^	Present Study

## Data Availability

The data presented in this study are available in the article.

## Supplementary Materials

The following supporting information can be downloaded at: https://www.mdpi.com/article/10.3390/polym17010039/s1. The details regarding the synthesis of polybenzoxazine-based carbon, the synthesis of amino cellulose, the instrumentation methods, and the characterization of amino cellulose using FT-IR and XPS, have been given in the supplementary information. Figure S1. FT-IR spectra of (a) cellulose, tosyl cellulose and amino cellulose and (b) hyaluronic acid and ACH. Figure S2. XPS spectrum of PBC-ACH showing the (a) survey spectrum (inset: elemental composition) and (b–d) deconvoluted spectra for C 1s, N 1s and O 1s.

## References

[1] Mutharani B., Ranganathan P., Chen S.M., Deepak Vishnu S.K. (2020). Stimuli-Enabled Reversible Switched Aclonifen Electrochemical Sensor Based on Smart PNIPAM/PANI-Cu Hybrid Conducting Microgel. Sens. Actuators B Chem..

[2] Stojanović Z., Durović A., Kravić S., Grahovac N., Suturović Z., Bursić V., Vuković G., Brezo T. (2016). A Simple and Rapid Electrochemical Sensing Method for Metribuzin Determination in Tap and River Water Samples. Anal. Methods.

[3] Ranganathan P., Mutharani B., Chen S.M., Sireesha P. (2019). Biocompatible Chitosan-Pectin Polyelectrolyte Complex for Simultaneous Electrochemical Determination of Metronidazole and Metribuzin. Carbohydr. Polym..

[4] Akdağ S., Sadeghi Rad T., Keyikoğlu R., Orooji Y., Yoon Y., Khataee A. (2022). Peroxydisulfate-Assisted Sonocatalytic Degradation of Metribuzin by La-Doped ZnFe Layered Double Hydroxide. Ultrason. Sonochem..

[5] Stenrød M., Perceval J., Benoit P., Almvik M., Bolli R.I., Eklo O.M., Sveistrup T.E., Kværner J. (2008). Cold Climatic Conditions: Effects on Bioavailability and Leaching of the Mobile Pesticide Metribuzin in a Silt Loam Soil in Norway. Cold Reg. Sci. Technol..

[6] Zagal J.H., Gulppi M.A., Depretz C., Lelièvre D. (1999). Synthesis and Electrocatalytic Properties of Octaalkoxycobalt Phthalocyanine for the Oxidation of 2-Mercaptoethanol. J. Porphyr. Phthalocyanines.

[7] Serelis K., Mantzos N., Meintani D., Konstantinou I. (2021). The Effect of Biochar, Hydrochar Particles and Dissolved Organic Matter on the Photodegradation of Metribuzin Herbicide in Aquatic Media. J. Environ. Chem. Eng..

[8] Antonopoulou M., Konstantinou I. (2014). Photocatalytic Treatment of Metribuzin Herbicide over TiO_2_ Aqueous Suspensions: Removal Efficiency, Identification of Transformation Products, Reaction Pathways and Ecotoxicity Evaluation. J. Photochem. Photobiol. A Chem..

[9] Pandiyan R., Vinothkumar V., Chen S.M., Sangili A., Kim T.H. (2023). Integrated LaFeO_3_/RGO Nanocomposite for the Sensitive Electrochemical Detection of Antibiotic Drug Metronidazole in Urine and Milk Samples. Appl. Surf. Sci..

[10] Benítez-Martínez S., López-Lorente Á.I., Valcárcel M. (2015). Multilayer Graphene-Gold Nanoparticle Hybrid Substrate for the SERS Determination of Metronidazole. Microchem. J..

[11] Jin W., Li W., Xu Q., Dong Q. (2000). Quantitative Assay of Metronidazole by Capillary Zone Electrophoresis with Amperometric Detection at a Gold Microelectrode. Electrophoresis.

[12] Orooji Y., Haddad Irani-nezhad M., Hassandoost R., Khataee A., Rahim Pouran S., Joo S.W. (2020). Cerium Doped Magnetite Nanoparticles for Highly Sensitive Detection of Metronidazole via Chemiluminescence Assay. Spectrochim. Acta Part A Mol. Biomol. Spectrosc..

[13] Daeseleire E., De Ruyck H., Van Renterghem R. (2000). Rapid Confirmatory Assay for the Simultaneous Detection of Ronidazole, Metronidazole and Dimetridazole in Eggs Using Liquid Chromatography-Tandem Mass Spectrometry. Analyst.

[14] Devasenathipathy R., Karuppiah C., Chen S.M., Mani V., Vasantha V.S., Ramaraj S. (2015). Highly Selective Determination of Cysteine Using a Composite Prepared from Multiwalled Carbon Nanotubes and Gold Nanoparticles Stabilized with Calcium Crosslinked Pectin. Microchim. Acta.

[15] Pasini Cabello S.D., Ochoa N.A., Takara E.A., Mollá S., Compañ V. (2017). Influence of Pectin as a Green Polymer Electrolyte on the Transport Properties of Chitosan-Pectin Membranes. Carbohydr. Polym..

[16] Pooja D., Panyaram S., Kulhari H., Rachamalla S.S., Sistla R. (2014). Xanthan Gum Stabilized Gold Nanoparticles: Characterization, Biocompatibility, Stability and Cytotoxicity. Carbohydr. Polym..

[17] Kadir M.F.Z., Hamsan M.H. (2018). Green Electrolytes Based on Dextran-Chitosan Blend and the Effect of NH_4_SCN as Proton Provider on the Electrical Response Studies. Ionics.

[18] Zhong X., Yuan R., Chai Y.Q. (2012). Synthesis of Chitosan-Prussian Blue-Graphene Composite Nanosheets for Electrochemical Detection of Glucose Based on Pseudobienzyme Channeling. Sens. Actuators B Chem..

[19] Rassas I., Braiek M., Bonhomme A., Bessueille F., Rafin G., Majdoub H., Jaffrezic-Renault N. (2019). Voltammetric Glucose Biosensor Based on Glucose Oxidase Encapsulation in a Chitosan-Kappa-Carrageenan Polyelectrolyte Complex. Mater. Sci. Eng. C.

[20] Shukur M.F., Ithnin R., Kadir M.F.Z. (2016). Ionic Conductivity and Dielectric Properties of Potato Starch-Magnesium Acetate Biopolymer Electrolytes: The Effect of Glycerol and 1-Butyl-3-Methylimidazolium Chloride. Ionics.

[21] Espíndola-González A., Martínez-Hernández A.L., Fernández-Escobar F., Castaño V.M., Brostow W., Datashvili T., Velasco-Santos C. (2011). Natural-Synthetic Hybrid Polymers Developed via Electrospinning: The Effect of PET in Chitosan/Starch System. Int. J. Mol. Sci..

[22] Kaushik A., Tiwari A., Gaur A. (2015). Role of Excipients and Polymeric Advancements in Preparation of Floating Drug Delivery Systems. Int. J. Pharm. Investig..

[23] Hsiao W.W.W., Lincy V., Selvi S.V., Prasannan A., Sambasivam S., Nimita Jebaranjitham J. (2024). Carrageenan Derived Polyelectrolyte Complexes Material: An Effective Bifunctional for Electrochemical Sensing of Sulfamethazine and Antibacterial Activity. Int. J. Biol. Macromol..

[24] Novotný V., Barek J. (2017). Voltammetric Determination of Aclonifen at a Silver Amalgam Electrode in Drinking and River Water. Ecol. Chem. Eng. S.

[25] Rodrigo M.A., Oturan M.A., Oturan N. (2014). Electrochemically Assisted Remediation of Pesticides in Soils and Water: A Review. Chem. Rev..

[26] Guziejewski D., Smarzewska S., Skowron M., Ciesielski W., Nosal-Wiercińska A., Skrzypek S. (2016). Rapid and Sensitive Voltammetric Determination of Aclonifen in Water Samples. Acta Chim. Slov..

[27] Inam R., Çakmak Z. (2013). A Simple Square Wave Voltammetric Method for the Determination of Aclonifen Herbicide. Anal. Methods.

[28] Thirukumaran P., Shakila Parveen A., Kim S.C. (2023). Heteroatom-Enhanced Porous Carbon Materials Based on Polybenzoxazine for Supercapacitor Electrodes and CO_2_ Capture. Polymers.

[29] Bigucci F., Luppi B., Cerchiara T., Sorrenti M., Bettinetti G., Rodriguez L., Zecchi V. (2008). Chitosan/pectin polyelectrolyte complexes: Selection of suitable preparative conditions for colon-specific delivery of vancomycin. Eur. J. Pharm. Sci..

[30] Maciel V.B.V., Yoshida C.M.P., Franco T.T. (2015). Chitosan/pectin polyelectrolyte complex as a pH indicator. Carbohydr. Polym..

[31] Liu Y., Wu T., Zhao H., Zhu G., Li F., Guo M., Ran Q., Komarneni S. (2023). An Electrochemical Sensor Modified with Novel Nanohybrid of Super-P Carbon Black@zeolitic-Imidazolate-Framework-8 for Sensitive Detection of Carbendazim. Ceram. Int..

[32] Shu X., Zhao H., Hu Y., Liu J., Tan M., Liu S., Zhang M., Ran Q., Li H., Liu X. (2018). Magnesium and Silicon Co-Doped LiNi_0.5_Mn_1.5_O_4_ Cathode Material with Outstanding Cycling Stability for Lithium-Ion Batteries. Vacuum.

[33] Zhao H., Li Y., Shen D., Yin Q., Ran Q. (2020). Significantly Enhanced Electrochemical Properties of LiMn2O4-Based Composite Microspheres Embedded with Nano-Carbon Black Particles. J. Mater. Res. Technol..

[34] Zhao H., Guo M., Li F., Zhou Y., Zhu G., Liu Y., Ran Q., Nie F., Dubovyk V. (2023). Fabrication of Gallic Acid Electrochemical Sensor Based on Interconnected Super-P Carbon Black@mesoporous Silica Nanocomposite Modified Glassy Carbon Electrode. J. Mater. Res. Technol..

[35] Zhao H., Zhu G., Li F., Liu Y., Guo M., Zhou L., Liu R., Komarneni S. (2023). 3D Interconnected Honeycomb-like Ginkgo Nut-Derived Porous Carbon Decorated with β-Cyclodextrin for Ultrasensitive Detection of Methyl Parathion. Sens. Actuators B Chem..

[36] Suea-Ngam A., Rattanarat P., Chailapakul O., Srisa-Art M. (2015). Electrochemical Droplet-Based Microfluidics Using Chip-Based Carbon Paste Electrodes for High-Throughput Analysis in Pharmaceutical Applications. Anal. Chim. Acta.

[37] Mutharani B., Rajakumaran R., Chen S.M., Ranganathan P., Tsai H.C. (2021). Hierarchical Polyacrylonitrile-Derived Nitrogen Self-Doped 3D Carbon Superstructures Enabling Electrochemical Detection of Calcium Channel Blocker Nimodipine in Real Human Blood Serum. ACS Sustain. Chem. Eng..

[38] Mutharani B., Ranganathan P., Yang J.M., Chang Y.H., Chiu F.C., Tsai H.C. (2022). Rational Assembly of Polymer-Metal Coordination Hierarchical Superstructures for Azathioprine-Responsive Electrodes in Biological Samples. ACS Appl. Nano Mater..

[39] Yao Y., Wen Y., Zhang L., Wang Z., Zhang H., Xu J. (2014). Electrochemical Recognition and Trace-Level Detection of Bactericide Carbendazim Using Carboxylic Group Functionalized Poly(3,4-Ethylenedioxythiophene) Mimic Electrode. Anal. Chim. Acta.

[40] Zhao H., Ma H., Li X., Liu B., Liu R., Komarneni S. (2021). Nanocomposite of Halloysite Nanotubes/Multi-Walled Carbon Nanotubes for Methyl Parathion Electrochemical Sensor Application. Appl. Clay Sci..

