# Supplementary material for: Electrochemical Sensing of Metribuzin Utilizing the Synergistic Effects of Cationic and Anionic Bio-Polymers with Hetero-Doped Carbon
Source: Polymers (Basel). 2024 Dec 27;17(1):39. doi: 10.3390/polym17010039 (PMC11723142; doi:10.3390/polym17010039)
Supplement: Supplementary file 1 [file polymers-17-00039-s001.zip › polymers-3340810-supplementary.pdf]

Supporting information

Electrochemical Sensing of Metribuzin Utilizing Synergistic Effects of Cationic and Anionic Bio-Polymers with Hetero-Doped Carbon

Thirukumaran Periyasamy[1†], Shakila Parveen Asrafali[1†], Seong-Cheol Kim[2] and Jaewoong Lee[1]*

[1] Department of Fiber System Engineering, Yeungnam University, Republic of Korea.

[2] School of Chemical Engineering, Yeungnam University, Republic of Korea.

[†] Authors contributed equally to this work.

* Author for correspondence.

Synthesis of polybenzoxazine based carbon (PBC)

Melamine (6.3 g) and formaldehyde (9 g) were placed in a 500 mL three-necked round-bottom flask equipped with a reflux condenser and magnetic stirrer. A mixture containing 200 mL of water and ethanol (1:1) was added and heated to 80 °C for 1 h. Separately, eugenol (24.6 g) was dissolved in 50 mL of the prepared water-ethanol mixture, and this solution was added dropwise to the reaction flask. The mixture was then refluxed at 100 °C for 12 h. After cooling to room temperature, the mixture was poured into 1 L of 1 M NaOH solution. The precipitate formed was filtered and washed several times with water. It was then dried in a vacuum oven at 60 °C, yielding a pale yellow benzoxazine powder. This benzoxazine monomer was cured by stepwise heating in an oven at 100, 150, 200, and 250 °C, each for an hour. The resulting polybenzoxazine (PBz) was carbonized at 800 °C under a nitrogen atmosphere for 5 h at a ramp rate of 1°C/min. At this temperature, only the carbon content of the polymer remains. The carbonized product was then treated in an aqueous KOH solution and heated to 120 °C to remove water. The activation process was conducted at 600 °C for 1 h in a tubular furnace under nitrogen flow with a ramp rate of 5 °C/min. The final material, named PBC, was obtained.

Synthesis of Amino Cellulose (AC)

Step 1: Preparation of tosyl cellulose (TC)

Initially, cellulose (5.0 g) was dissolved in 150 mL of N, N-dimethylacetamide (DMAc). The mixture was then heated and stirred continuously at 120 °C for 1 h to ensure complete dissolution. After this period, 8.0 g of anhydrous lithium chloride (LiCl) was gradually added to the solution while stirring was maintained. This step was continued until a clear, viscous solution was formed, indicating the complete dissolution of cellulose in the DMAc/LiCl solvent system. Separately, a solution of triethylamine (17.3 mL) in DMAc (15 mL) was prepared and cooled to 8 °C. Subsequently, a solution of tosyl chloride (11.9 g) in 25 mL of DMAc was added slowly to this mixture while maintaining the low temperature. The resulting solution was then added dropwise to the homogenous cellulose solution under continuous stirring at 8 °C. During this process, the initial pale-yellow solution turned dark reddish-brown, which suggested the successful tosylation of cellulose. The reaction was allowed to proceed for 24 h under the same conditions. After the completion of the reaction, the tosylated cellulose (TC) was precipitated by pouring the reaction mixture into 1500 mL of ice-cold water. The precipitated product was then collected by filtration, thoroughly washed with deionized water and ethanol to remove any unreacted reagents, and subsequently dried under vacuum at 50 °C. The resulting product, p-tosyl cellulose (TC), was obtained as a purified solid.

Step 2: Amination to Form Amino Cellulose (AC)

In the next step, tosyl cellulose (2.0 g) was suspended in dimethyl sulfoxide (DMSO), followed by the addition of ethylene diamine (9 g). The mixture was then heated to 100 °C and stirred continuously for 5 h to facilitate the substitution of tosyl groups with amino groups. This amination process leads to the formation of amino cellulose (AC). After the reaction was complete, the

mixture was allowed to cool to room temperature. The resultant polymer, amino cellulose, was precipitated by adding the reaction mixture into acetone. The precipitated amino cellulose was then collected by filtration and washed sequentially with acetone and ethanol to remove any residual solvent and by-products. The final product was then dried under vacuum at 50 °C to yield amino cellulose (AC) in solid form. Through this two-step process—tosylation of cellulose followed by amination, a successful conversion of cellulose to amino cellulose (AC) was achieved.

Instrumentation methods

The surface morphology and chemical composition were studied by FESEM with EDS analysis was carried out on a Hitachi S-4800 equipped with EDX at an accelerating voltage of 4 kV. TEM/HRTEM images were performed with an FEI-Tecnai TF-20 transmission electron microscope with an operating accelerating voltage of 120 kV. ATR-FTIR spectra were recorded in transmittance mode on a Perkin Elmer Spectrum Two in the wavenumber range from 400 to 4000 cm^{-1} by the addition of 16 scans at a resolution of 16 cm^{-1}. XPS spectra were achieved using a K-Alpha (Thermo Scientific). CasaXPS software was used for the deconvolution of the high-resolution XPS spectra. Electrocatalytic performance was investigated through cyclic voltammetry (CV) and linear sweep voltammetry (LSV) using CORRTEST. In this study, a conventional three-electrode system was employed, including prepared materials GCE as the working electrode, a saturated Ag/AgCl as the reference electrode, and a Pt wire as the counter electrode.

Characterization of amino cellulose (AC)

Cellulose was converted to amino cellulose by replacing its hydroxyl groups with amino groups. This substitution was achieved through a nucleophilic substitution reaction, where tosyl cellulose served as an intermediate, as illustrated in Scheme1. The chemical structure of amino cellulose (AC) was analyzed using FT-IR (Fourier-transform infrared) spectroscopy. Figure S1a

presents the FT-IR spectra for cellulose, tosyl cellulose, and amino cellulose. In the FT-IR spectrum of cellulose, characteristic absorption peaks appear at 3300, 1582, 1412, 1071, and 890 cm^{-1}. These peaks correspond to the stretching or bending vibrations of the -OH, -CH, absorbed water, $-CH_2$, C-O-C, and β-glycosidic bonds, respectively. When examining the FT-IR spectrum of tosyl cellulose, additional peaks were observed at 1586, 1502, 1483, and 801 cm^{-1}, which are attributed to a p-substituted benzene ring, while peaks at 1373 and 1165 cm^{-1} are related to the O-S-O group. For amino cellulose, the FT-IR spectrum displays new peaks at 1594, 1458, and 1314 cm^{-1}, which are indicative of C–N, $-NH_2$, and –NH groups, respectively. Additionally, a peak at 1152 cm^{-1} suggests the presence of C−N−C symmetric stretching vibrations. The notable peak at 1594 cm^{-1} is specifically linked to the amino group from ethylene diamine. Meanwhile, the methylene groups ($-CH_2$) from ethylene diamine exhibit stretching vibrations between 2916 and 2828 cm^{-1}. These spectra provide clear evidence of the successful conversion of cellulose to amino cellulose.

The FTIR spectra of the produced ACH (Fig. S1b) closely resembles that of the hyaluronic acid. The prominent band near 3500 cm^{-1} is associated with the O-H and N-H stretching vibrations of the N-acetyl side chain, indicative of hydrogen bonding. Around 2928 cm^{-1}, a cluster of moderate-intensity overlapping bands appears, corresponding to C-H stretching vibrations. The bands at 1624 and 1420 cm^{-1} are linked to the asymmetric (C=O) and symmetric (C-O) stretching vibrations of the planar carboxyl groups in hyaluronic acid. When amino cellulose is blended with hyaluronic acid, the resulting FTIR spectrum is a combination of the characteristic peaks from both substances, potentially showing shifts due to interactions like hydrogen bonding. The peak at 3500 cm^{-1} is more intensified and broadened due to hydrogen bonding interactions between -OH and -NH groups. C-H stretching is also observed at 2924 cm^{-1}. Similarly, there is a shift in the C=O stretching peaks, typically between 1600-1750 cm^{-1}, possibly due to the interaction between

the amine groups of amino cellulose and the carboxyl groups of hyaluronic acid. N-H bending vibrations of the amine groups was observed around 1623 cm^{-1}, while C-O-C stretching (ether linkages) appears near 1052 cm^{-1}. The obtained results confirm the successful synthesis of amino ellulose and ACH.

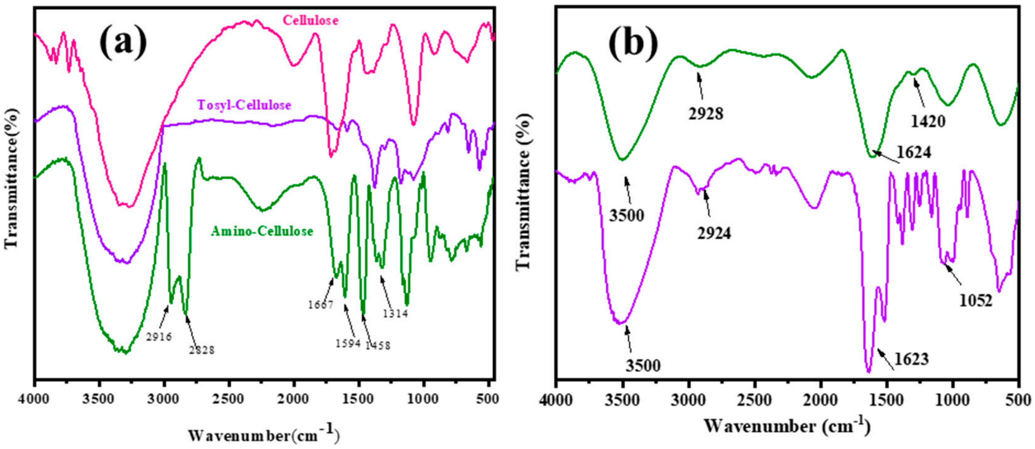

Figure S1: FT-IR spectra of (a) cellulose, tosyl cellulose and amino cellulose and (b) hyaluronic acid and ACH.

XPS analysis

Figures S2a-d displays X-ray Photoelectron Spectroscopy (XPS) data for PBC-ACH. XPS is a technique that provides information about the surface composition, chemical states, and electronic states of elements in a material. The four subplots in the figure represent different aspects of the analysis. Figure S2a shows a survey scan, offering a general overview of the elements detected on the ACH-coated PBC surface. Peaks identified as C 1s, O 1s, and N 1s correspond to the presence of carbon (C), oxygen (O), and nitrogen (N), indicating these elements are present on the surface of the material. The intensity of each peak is related to the relative

abundance of these elements. The highest peak intensity of carbon is the expected one, as the sample is based on carbon material coated with ACH layer. The inset pie chart in this plot shows the atomic percentages of the elements detected. The dominant percentage of carbon suggests that it is the primary component, followed by oxygen and nitrogen. Figure S2b plot shows a high-resolution scan of the C 1s region. This spectrum is deconvoluted into multiple peaks that correspond to different chemical states of carbon. The peaks at different binding energies can be attributed to various carbon species. C-C or C-H (~284.5 eV): Represents carbon in aliphatic or aromatic structures, which is common in carbon materials. C-N (~286 eV): Indicates carbon bound to nitrogen, which is attributed to the amino groups from the ACH coating. C-O-C (~286.5 eV): Corresponds to ether groups, likely from the ACH structure. C=O (~288 eV): Represents carbon in carbonyl groups, which could be from oxidation of the hyaluronic acid, producing defects in the carbon structure. The presence of these various carbon species confirms the successful coating of the carbon material with ACH, as it introduces C-N and C-O-C functionalities. Figure S2c displays a high-resolution N 1s spectrum, showing different nitrogen states present in the sample. The deconvolution of the spectrum shows peaks corresponding to different types of nitrogen. Graphitic N (~400 eV): Indicates nitrogen bonded within the graphitic lattice, resulting from nitrogen incorporation during the synthesis of carbon material. Pyridinic N (~398.5 eV) and Pyrrolic N (~401 eV): These species are typically found in nitrogen-doped carbon materials, showing that the nitrogen is incorporated into the carbon matrix. The appearance of these nitrogen states suggests that nitrogen may be partially embedded within the carbon structure and partially from the ACH coating. Figure S2d illustrates the high-resolution O 1s spectrum. The deconvolution shows peaks that correspond to different oxygen functionalities. C=O (~531.5 eV): Indicates carbonyl oxygen, likely from oxidized groups in cellulose or carbon material. C-O (~533 eV): Represents ether and

alcohol groups, which can be attributed to the cellulose structure. COO^- (~532.5 eV): Indicate carboxyl groups resulting from hyaluronic acid. The deconvoluted XPS peaks provide insight into the specific bonding states, confirming the successful coating of PBC material with ACH. The chemical state distribution of C 1s, N 1s, and O 1s peaks indicates the incorporation of different functional groups, which could be beneficial for specific applications such as catalysis, adsorption, or as a support material for various chemical processes.

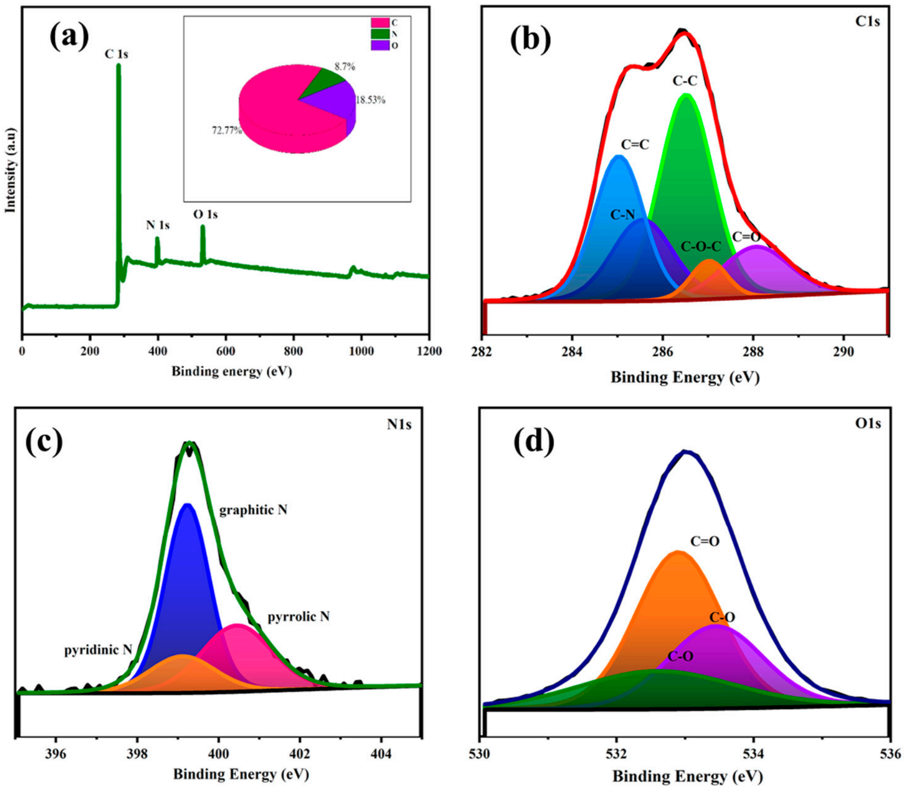

Figure S2: XPS spectrum of PBC-ACH showing the (a) survey spectrum (inset: elemental composition) and (b-d) deconvoluted spectra for C 1s, N 1s and O 1s.
